# A Mendelian randomization study of genetic liability to cutaneous melanoma and sunburns

**DOI:** 10.3389/fonc.2024.1393833

**Published:** 2024-08-30

**Authors:** Fengmin Lu, Ling Wang, Xixing Ma, Yanling Li

**Affiliations:** Department of Dermatology, Clinical Medical Research Center of Dermatology and Venereal Disease in Hebei Province, The Second Hospital of Hebei Medical University, Shijiazhuang, China

**Keywords:** cutaneous melanoma, sunburns, Mendelian randomization study, genetic liability, dermatology

## Abstract

**Background:**

Some studies have reported that sunburns and cutaneous melanoma (CM) risk is increasing, but a clear causal link has yet to be established.

**Methods:**

This current study conducted a two-sample Mendelian randomization (MR) approach to clarify the association and causality between sunburn history and CM using large-scale genome-wide association study data.

**Results:**

The inverse-variance weighted method result showed that sunburn might be associated with the risk of CM increasing (p = 2.21 × 10^−23^, OR = 1.034, 95% CI= 1.027-1.041), causally. The MR-Egger regression, weighted median method, simple mode method, and weighted mode method results showed similar results.

**Conclusion:**

This study offers evidence of sunburn history and increased risk of CM, and it shows that there might be common genetic basics regarding sunburns and CM susceptibility in Caucasian, European, or British ethnic groups.

## Introduction

Cutaneous melanoma (CM) originates from melanocytes located in the basal layer of the skin’s epidermis. It accounts for 5%~10% of all skin cancers yet accounts for over 80% of skin cancer-related deaths (1, 2) Yuan et al. reported that CM prevalence increased in most countries between 1990 and 2019, and the predictive analysis results suggested a declining trend in the age-standardized incidence rate incidence but a growing number of CM (3). Arnold et al. estimated that there might be a total of 325,000 new cases and 57,000 deaths from CM worldwide in 2020; new cases would increase to 510,000 (a roughly 50% increase) and 96,000 deaths (a 68% increase) by 2040 (4)]. The treatment of CM includes wide excision, checkpoint immunotherapy, targeted therapy, and radiotherapy, and multidisciplinary care models have been widely used in advanced CM (5). Immunotherapy has significantly improved the outcome of CM, but its expensive price has become an important factor hindering the survival rate of low-income CM patients (5–7).

CM is a result of the complex interaction between genetic and environmental risk factors. Sunburns have been identified as the first risk factor for CM (1, 2, 8). An Australian study showed that sunscreen could effectively reduce the incidence of CM (9). Most studies have focused on the association between URV exposure and CM, but the causal relationship between sunburn history (severe URV exposure history) and CM has often been overlooked.

We conducted a two-sample Mendelian randomization (MR) study to clarify the association and causality between sunburn history and CM using large-scale genome-wide association studies (GWAS) data. This study provides new perspectives and evidence for the early prevention and potential pathogenesis of CM.

## Methods

### Data sources and instrumental variable selection

The data deployed in this study were publicly available GWAS datasets validated by the IEU open GWAS database (https://gwas.mrcieu.ac.uk), so this study did not require ethical approval.

The GWAS dataset of sunburn (ebi-a-GCST90029034) was released in 2018 (10), including 350,232 participants. The GWAS dataset of CM (ieu-b-4969) was released in 2021, including 375,767 participants. The population of exposure factors we selected were 350,232 European ancestry individuals, mainly from the United Kingdom, European (U.K.); the population of melanoma was mainly from the UK Biobank, which is European British ethnic. Based on the utilization of aggregated-level data in our research, which lacks specific biological individual information, and following the data usage policy of the UKBB GWAS results, no further authorization was necessary for the utilization of UK biological database data in this study (https://www.nealelab.is/uk-biobank/faq).

Sunburn-related SNPs were considered to be instrumental variables (genome-wide significance: p < 5 × 10^−8^; clumping algorithm: r^2^ = 0.001 and kb = 10,000) (11). F statistics of ≥10 demonstrated a low risk of weak instrumental bias (12, 13).

### MR analyses

A two-sample MR strategy was used in this study, and SNP exposure and SNP outcome associations were estimated using summary statistics from independent samples. We utilized five MR methods to obtain the causality between sunburns and CM: MR-Egger regression (14), weighted median method (15), inverse-variance weighted (IVW) (16), simple mode, and weighted mode. The IVW method uses a weighted linear regression (Wald ratio of each SNP) of SNP-exposure coefficients and SNP-disease coefficients to estimate the effect of exposure on outcomes. This method minimizes the mean variance and is often used in meta-analyses to integrate independent measurement results. The weighted median estimation provides accurate estimates if at least half of the IVs are valid. The MR-Egger regression assumes that all IVs are invalid and is the least powerful in causality inference. It typically serves to validate the direction of the effects.

**Figure 1:**
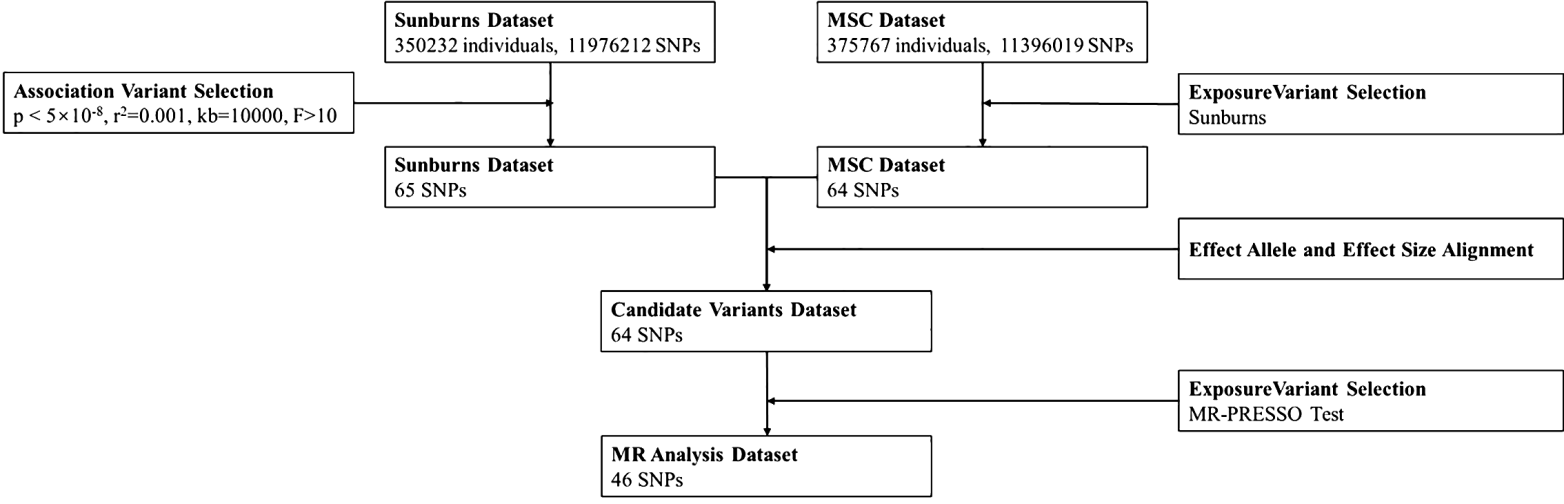
Flow of the MR study.

MR study results with p < 0.05 were considered significant. Effect sizes are provided as odds ratio (OR) alongside 95% confidence intervals (CIs). All MR analyses were performed in R (Version 4.3.1) using the TwoSampleMR package (version 0.5.0) (17). **Figure 1** shows the flow of the MR study.

### Sensitivity analyses and power calculation

To evaluate the reliability of our results, the Cochran Q-test was performed to confirm the statistical heterogeneity of SNPs using IVW estimates. p < 0.05 was considered as the sign of sign of significant heterogeneity. Heterogeneity was judged visually by funnel plots. Horizontal pleiotropy was evaluated using the MR-Egger intercept term (18), and an MR-PRESSO test (19) was used in a leave-one-out (LOO) analysis to exclude each SNP in turn and repeated IVW estimation.

The power calculation for the IVW estimates was based on mRnd (20) (https://shiny.cnsgenomics.com/mRnd/). Sufficient statistical power is recommended to be over 80%.

## Results

### Mendelian randomization analysis

At beginning, we used a total of 56 snps (instrumental variables) according to the threshold setting in the method. After controlling confounding factors by MRlap package and MR-PRESSO outlier test, we identified 49 SNPs for the MR analysis. The F statistics were above 10 (ranging from 27.088 to 1,463.858, with an average of 111.274) (**Supplementary Table 1**). The results showed that the possibility of weak instrument bias might be ruled out (21).

In addition, MR-PRESSO outlier test is used to eliminate outliers and to remove horizontally pleiotropic SNPs. By MR-PRESSO outlier test, we deleted rs12203592, rs3114908, rs4406278, rs62209647, etc. The corrected MR results still significant (**Supplementary Table 2**).

**Figure 2:**
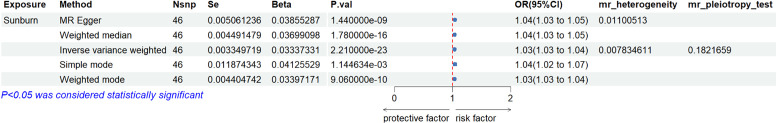
Forest plot to visualize the causal effect of sunburn on the risk of MCS using all MR methods.

**Figure 3:**
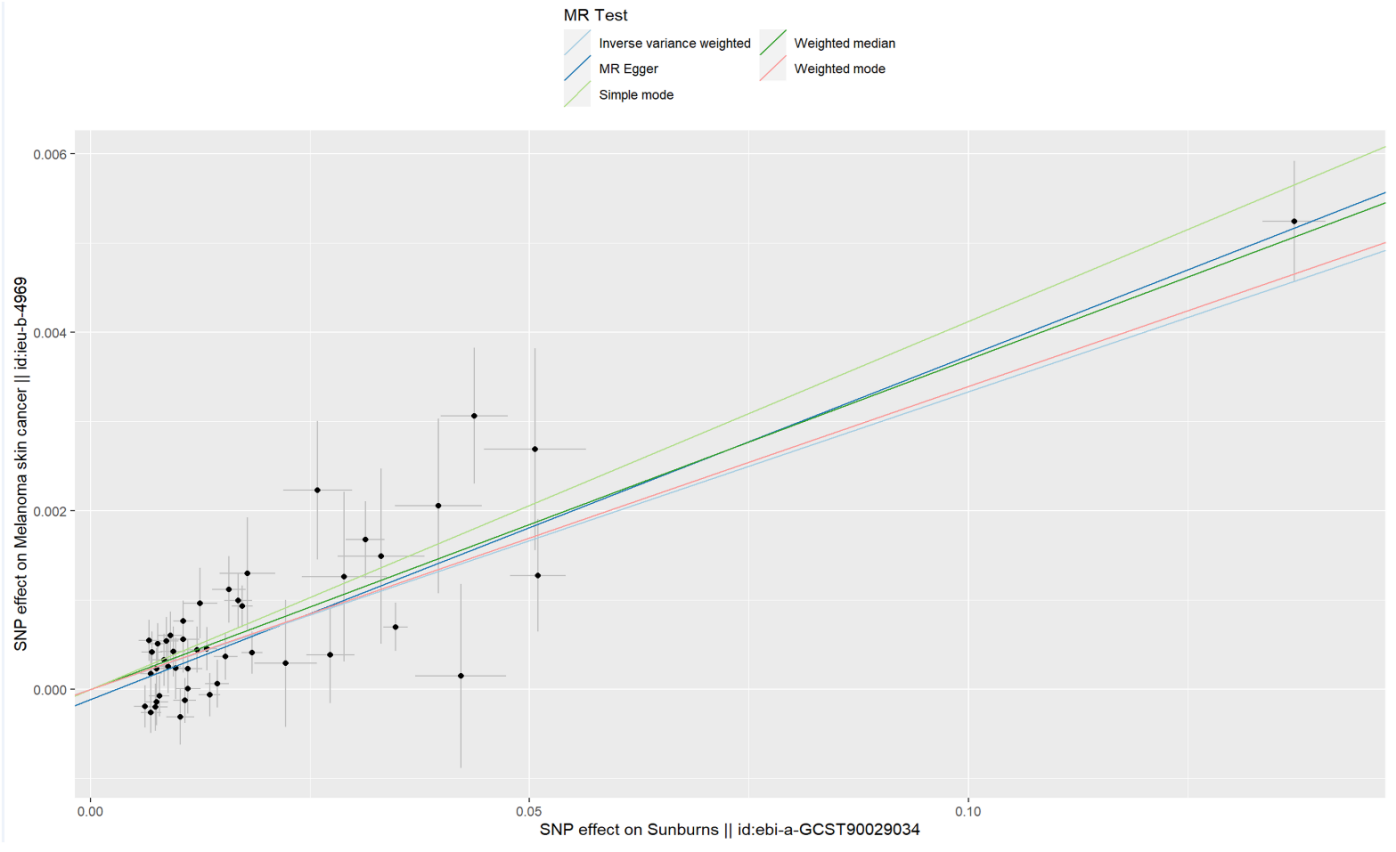
Scatter plot to visualize the causal effect of sunburn on the risk of MCS. The slope of the straight line indicates the magnitude of the causal association.

The results of the IVW method suggested that sunburn might be associated with the risk of CM increasing (p = 2.21 × 10^−23^, OR = 1.034, 95% CI = 1.027–1.041), causally. Also, the other four MR method analysis results showed similar results (**Table 1**, **Figure 2**). As the risk of sunburn rises, so does the risk of CM (**Figure 3**).

**Table 1:** Association between sunburn and risk of melanoma skin cancer.

Method	Beta	SE	*p*	OR (95% CI)
MR-Egger	0.039	0.005	1.44×10^9^	1.039 (1.029-1.050)
Weighted median	0.037	0.004	1.78×10^−16^	1.038 (1.029-1.047)
Inverse variance weighted	0.033	0.003	2.21×10^−23^	1.034 (1.027-1.041)
Simple mode	0.041	0.012	1.15×10^−3^	1.042 (1.018-1.067)
Weighted mode	0.034	0.004	9.06×10^−10^	1.035 (1.026-1.044)

Due to the heterogeneity of the data, we finally used two methods, fixed effects and random effects models, to calculate the Mendelian analysis result. The results of all six methods are all in the same direction, indicating that they are harmful factors, and the p values of all methods show a high degree of correlation. The fixed effects model p-value was 5.541543e−51 (**Supplementary Table 3**), still significant either. The power calculation for the IVW estimates based on mRnd was over 80% sufficient for the statistics.

### Sensitivity analyses

**Figure 4:**
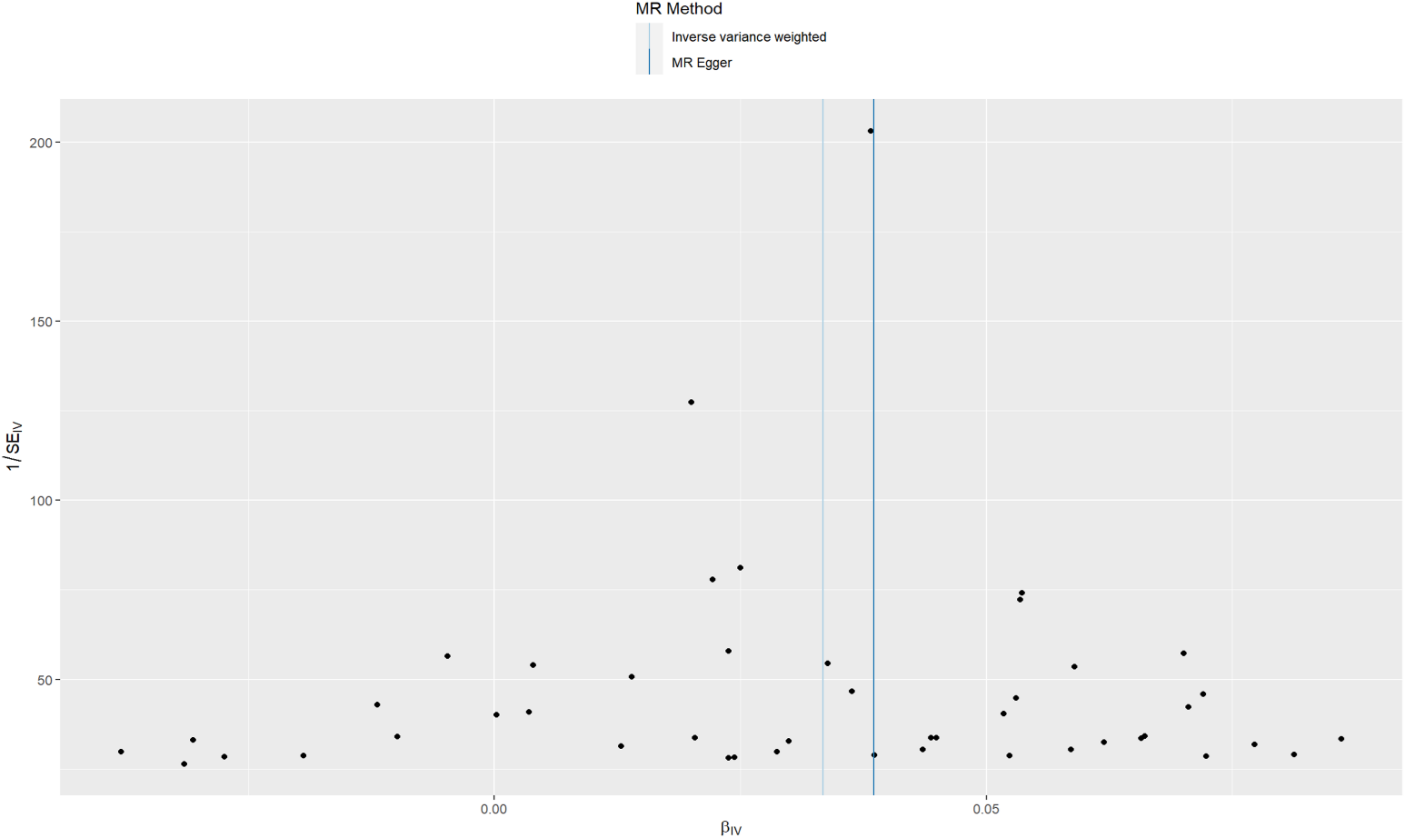
Funnel plots to visualize the overall heterogeneity of MR estimates for the effect of sunburn on CM.

Sensitivity analyses were performed to confirm whether the association was obtained through the MR assumptions violation. The results showed that there was no heterogeneity (I^2^ = 0.01.1). There was no evidence suggesting that directional pleiotropy caused risk estimates (IVW MR_pleiotropy_test p = 0.182). An MR funnel plot was used in the visual assessment of directional pleiotropy, and the results showed that all variants were distributed symmetrically (**Figure 4**), suggesting that estimates might not be caused by unknown outliers.

**Figure 5:**
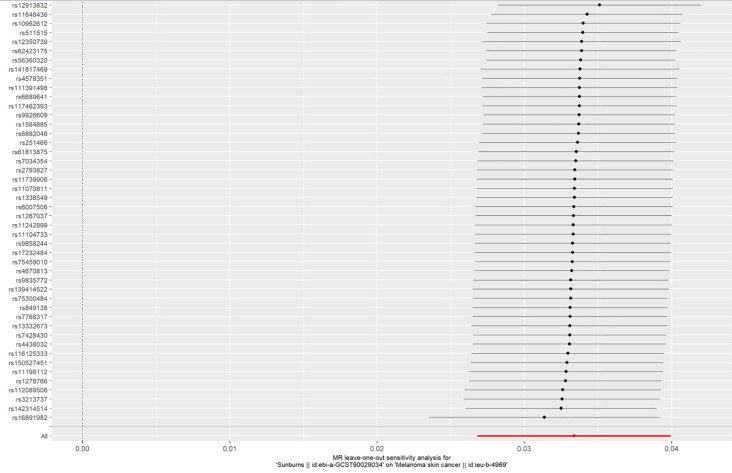
LOO plot to visualize the causal effect of sunburn on the risk of CM when one SNP is left out.

A LOO sensitivity analysis was performed using the MR-PRESSO test. The results suggest that there might be no single-SNP-caused bias (**Figure 5**).

## Discussion

The direct cause of CM is the long-term accumulation of mutations in skin cells. To determine the causal relationship between sunburns and CM, long-term continuous follow-up and experimental studies based on basic medicine are required. Due to high costs, including the ongoing investment of money and time, a long-term randomized cohort study is a very challenging job. This limits our ability to obtain a more complete picture of CM risk factors and the corresponding causal relationships between sunburn history and CM. In general, germline genetic variants could not be affected by environmental risk factors because their combined form had already been determined at the time of zygote formation. This statute can be approximated as completely randomized, comparable with the design principles used in randomized controlled trials (22). With germline genetic variants as proxies for risk factors, through the reanalysis of GWAS data, MR analysis can replace long-term randomized cohort studies to some extent. Thus, the MR study could also effectively address the issue of reverse causality.

The results of the MR study, which involved a combined sample of 3,751 cases and 372,016 non-cases, revealed an association between sunburn history and increased CM risk. A comprehensive meta-analysis reported that intermittent sun exposure and sunburn history increased the risk of CM (23). A systematic review of gene–sun exposure interactions in skin cancer identified variants of *MC1R*, *CAT*, and *NOS1* that could help explain the mechanism by which sun exposure causes CM (24).

There have been three MR studies about sunburns and CM, and we obtained similar results. Li et al. reported that childhood sunburn and malignant melanoma were indicated (OR = 4.74), sites of tumor occurrence including on the face and trunk (25). Zhong et al. reported that elevating the number of sunburns during childhood increased the risk of CM (26). Liu et al. reported that a history of childhood sunburn might increase the risk of CM (OR = 6.317) (12). Data sources of sunburns for the above three studies were obtained from the UK Biobank, but melanoma data were obtained from the FinnGen Biobank and 23andMe. Although these databases are derived from European populations, the influence of genetic backgrounds between the British and Finnish populations cannot be ruled out. The data in this study are from the UK Biobank; the population involved in the study is British, which can partly reduce the influence of the genetic backgrounds of different populations on the MR results.

Previous evidence suggests that the PI3K/Akt and MAKP pathways might play important roles in UVR-related CM (27). However, this mechanism cannot explain all the etiology of CM. The melanoma GWAS meta-analyses by Landi et al. reported 54 genome-wide significant loci (28); based on the result, Erping Long identified functional variants and target genes for melanoma (29). We found overlapping genes in sunburn-associated variants (which we selected in this study) and in the melanoma susceptibility gene, as reported in the above two studies. The overlap gene set includes SLC45A2, HAL, and CYP1B1, whose related SNP set contains rs16891982, rs3213737, and rs4670813. rs16891982, a locus in SLC45A2, has been reported as a skin ageing- (30), skin pigmentation- (31, 32), and skin photosensitivity- (33) associated SNP.

Previous studies about HAL, containing the rs3213737 locus, might play roles in basal cell carcinoma (24) and skin pigmentation (34). However, there had been no study that reported the association between rs4670813 and CM, and CYP1B1 whose rs4670813 locus might be involved in the occurrence and progression of CM (35–37). This result suggests that there might be a common occurrence basis, including common genes and mechanisms. An in-depth analysis of the mechanism would help to comprehensively understand the relationship between sunburn history and CM. This relationship not only is epidemiological but can also reveal the specific biological process.

Cutaneous malignant melanoma is skin tumor which accounts for the third place among skin malignant tumors (approximately 6.8%~20%) (38). The conclusion of our study suggests sunburn harmful for CM, so the following intervention strategies and related research have positive healthy implications. Preventive strategies are as follows: avoiding overexposure to the sun and taking protective measures such as using sunscreen, wearing a sunhat, and clothing can help reduce the risk of melanoma. Melanoma is highly hereditary and family clustering. The high incidence of family aggregation can detect the CM early, and this can be an early warning method for whole family screening (39). For prognostic screening testing for melanoma: Prognostic tests can be used to estimate the severity of a melanoma and can help inform further treatment decisions. Such as UCSF 500 test and Gene Expression Profiling (GEP) which are relatively new and have not been universally adopted (39–41). Early treatment: The prognosis of early complete surgical resection, maturity of immunotherapy, and targeted therapy technology all have good short-term and long-term effects with few side effects.

This MR analysis presented some limitations in data availability. It is inevitable that there will be some overlap, which may lead to bias and confounding. In addition, sun exposure is not the only factor causing melanoma. The influence of other confounding factors needs to be analyzed in future: the elderly people and people with fair skin, with acral skin nevus, and with a history of melanoma or skin diseases are all high-risk groups for melanoma (38–40). The age of the population in this study (UK Biobank) is the British population aged 40–69, so the analysis results may be confounded by age factors. Many confounding factors may affect the results of our analysis. However, the sensitivity test is reliable (**Supplementary Tables 1**–**3**), so the confounding factors did not affect our results.

## Conclusion

This two-sample MR study provided a positive correlation between the number of sunburns and increased CM risk. Sunburn’s genetic susceptibility might lead to CM risk. This suggests that people with a history of sunburns should be on high alert for the occurrence of CM. The scope of application of the conclusion in our analysis is limited to Caucasian, European, or British ethnic groups. Whether there is significance in Asian and other races is yet to be studied. Public health and clinical departments should pay more attention to the prevention of sunburn to reduce the occurrence of CM.

## Data Availability

The original contributions presented in the study are included in the article/**Supplementary Material**. Further inquiries can be directed to the corresponding author.
