# Supplementary material for: A Mendelian randomization study of genetic liability to cutaneous melanoma and sunburns
Source: Front Oncol. 2024 Aug 30;14:1393833. doi: 10.3389/fonc.2024.1393833 (PMC11392754; doi:10.3389/fonc.2024.1393833)
Supplement: Supplementary file 1 [file DataSheet1.docx]

supplementary table1 Association between SNPs and sunburn

| SNP | effect allele.exposure | other allele.exposure | beta.exposure | se.exposure | pos.exposure | pval.exposure | F statistic | R^2^（%） |
| --- | --- | --- | --- | --- | --- | --- | --- | --- |
| rs10962612 | G | T | 0.0144256 | 0.00136487 | 16804167 | 6.30E-26 | 111.7082364 | 0.0757 |
| rs11070811 | T | C | -0.00882261 | 0.00150395 | 31394082 | 5.10E-09 | 34.41338297 | 0.0233 |
| rs11104733 | T | C | 0.0289156 | 0.00484078 | 88490119 | 2.40E-09 | 35.68072954 | 0.0243 |
| rs111391498 | G | A | -0.0273307 | 0.00271675 | 1341553 | 6.90E-24 | 101.2050442 | 0.0684 |
| rs11198112 | T | C | -0.0168093 | 0.00158946 | 119564143 | 2.30E-26 | 111.8407448 | 0.0779 |
| rs112089506 | T | C | -0.0313021 | 0.00218328 | 90149171 | 2.00E-47 | 205.5550147 | 0.1464 |
| rs11242899 | A | G | -0.0121293 | 0.00131863 | 460302 | 1.10E-19 | 84.6107552 | 0.0575 |
| rs116125333 | G | T | -0.0258831 | 0.00393365 | 33930012 | 2.50E-10 | 43.29533949 | 0.0303 |
| rs11648436 | T | C | -0.0135774 | 0.0012093 | 14008674 | 7.90E-31 | 126.0564638 | 0.0845 |
| rs11739906 | C | A | 0.00690311 | 0.0012387 | 59019359 | 2.70E-08 | 31.05684342 | 0.0210 |
| rs117462393 | T | C | 0.0421824 | 0.00519637 | 14277804 | 5.70E-16 | 65.89651356 | 0.0506 |
| rs12092858 | T | A | 0.00777025 | 0.00116723 | 73678454 | 4.00E-11 | 44.31564775 | 0.0301 |
| rs12350739 | A | G | 0.0184066 | 0.00120001 | 16885017 | 8.30E-55 | 235.2758867 | 0.1615 |
| rs1267037 | C | A | -0.00842275 | 0.00148486 | 162098155 | 4.10E-08 | 32.17635201 | 0.0222 |
| rs1278766 | C | T | 0.0105867 | 0.00117015 | 113534382 | 2.20E-20 | 81.85366675 | 0.0556 |
| rs12913832 | G | A | 0.0347646 | 0.00140463 | 28365618 | 1.50E-138 | 612.5627643 | 0.4180 |
| rs13332673 | T | G | -0.0506594 | 0.00579333 | 89940386 | 1.20E-18 | 76.46515066 | 0.0512 |
| rs1338549 | G | T | -0.0075531 | 0.00116776 | 98312143 | 4.40E-11 | 41.83533772 | 0.0284 |
| rs1391659 | C | G | 0.00695191 | 0.00116512 | 155984399 | 8.30E-10 | 35.60139106 | 0.0242 |
| rs139414522 | C | T | -0.039621 | 0.00493956 | 89821155 | 5.30E-16 | 64.33900421 | 0.0473 |
| rs141817469 | T | C | -0.0509909 | 0.00317023 | 89927151 | 2.50E-58 | 258.7043981 | 0.1762 |
| rs142314514 | G | A | -0.0436983 | 0.00382382 | 90130996 | 8.00E-31 | 130.5973014 | 0.1060 |
| rs150527451 | A | G | 0.0157916 | 0.00189641 | 68817897 | 2.60E-16 | 69.34063208 | 0.0475 |
| rs1584885 | T | G | -0.00618442 | 0.00118826 | 44647038 | 3.40E-08 | 27.08787872 | 0.0183 |
| rs16891982 | G | C | 0.137113 | 0.00358368 | 33951693 | 1.00E-200 | 1463.857562 | 0.9907 |
| rs17232484 | A | G | 0.0330942 | 0.0049553 | 89861650 | 4.30E-12 | 44.60297939 | 0.0305 |
| rs251466 | G | C | -0.0154047 | 0.00135208 | 149195603 | 5.90E-31 | 129.8080759 | 0.0886 |
| rs2793827 | T | A | -0.00969635 | 0.00175008 | 120448805 | 3.60E-08 | 30.69734135 | 0.0207 |
| rs3213737 | A | G | -0.0172716 | 0.00118316 | 96379806 | 8.50E-50 | 213.0974169 | 0.1457 |
| rs4438032 | G | C | 0.0124656 | 0.00199637 | 88786692 | 1.40E-10 | 38.98919819 | 0.0263 |
| rs4578351 | C | T | -0.0111169 | 0.00140868 | 16587580 | 2.70E-15 | 62.27915347 | 0.0426 |
| rs4670813 | A | G | -0.00702084 | 0.00117199 | 38317710 | 7.70E-09 | 35.88643685 | 0.0246 |
| rs511515 | G | A | -0.0107611 | 0.0012889 | 33541507 | 3.80E-17 | 69.7067584 | 0.0486 |
| rs56360320 | C | A | -0.00690393 | 0.00117413 | 189209646 | 1.40E-09 | 34.57485673 | 0.0234 |
| rs6007506 | T | C | -0.0132534 | 0.00123454 | 45622014 | 7.10E-27 | 115.2508877 | 0.0785 |
| rs61813875 | G | C | 0.0222366 | 0.0035909 | 152536650 | 5.60E-11 | 38.34688984 | 0.0290 |
| rs62423175 | A | G | -0.0102294 | 0.00156588 | 62195368 | 9.50E-11 | 42.67596674 | 0.0303 |
| rs6689641 | G | A | 0.00784297 | 0.0011681 | 110720400 | 7.50E-12 | 45.08178046 | 0.0305 |
| rs6882046 | G | A | 0.00750885 | 0.00132661 | 87968864 | 2.00E-09 | 32.03762577 | 0.0221 |
| rs7034354 | G | A | 0.0110752 | 0.00167262 | 12732575 | 1.10E-10 | 43.84384001 | 0.0299 |
| rs7428430 | T | C | 0.00666553 | 0.00116685 | 50174184 | 1.40E-08 | 32.63167101 | 0.0222 |
| rs75300484 | T | C | -0.0178944 | 0.0031568 | 89489061 | 1.20E-08 | 32.13217668 | 0.0215 |
| rs75459010 | T | C | 0.0105765 | 0.0018643 | 88017456 | 2.90E-08 | 32.18491365 | 0.0229 |
| rs7768317 | T | C | -0.00909163 | 0.00134734 | 41922220 | 2.80E-11 | 45.53329713 | 0.0309 |
| rs849138 | A | G | 0.00769742 | 0.00116479 | 28177338 | 1.90E-10 | 43.67119752 | 0.0296 |
| rs9835772 | T | A | 0.00866711 | 0.00135497 | 85766025 | 1.30E-10 | 40.91563141 | 0.0277 |
| rs9858244 | A | G | 0.00943232 | 0.00142087 | 85787399 | 4.60E-11 | 44.0685096 | 0.0299 |
| rs9904468 | G | C | 0.00802323 | 0.0011657 | 17573187 | 2.90E-11 | 47.37234587 | 0.0320 |
| rs9926609 | C | A | -0.00743547 | 0.00131994 | 3540962 | 2.50E-08 | 31.7328056 | 0.0215 |
| Average of the F statistics | |  |  |  |  |  | 111.2745673 |  |
| Summary of R^2^（%） | |  |  |  |  |  |  | 3.747 |

supplementary table2 MR-PRESSO Outlier Test results

|  | MR-PRESSO Outlier Test | |
| --- | --- | --- |
| SNP | RSSobs | Pvalue |
| rs10962612 | 1.03E-07 | 1 |
| rs11070811 | 6.05E-10 | 1 |
| rs11076692 | 1.32E-09 | 1 |
| rs11104733 | 2.60E-07 | 1 |
| rs111391498 | 1.09E-07 | 1 |
| rs11198112 | 3.14E-07 | 1 |
| rs11210187 | 8.89E-08 | 1 |
| rs11242899 | 1.62E-08 | 1 |
| rs11264244 | 1.31E-06 | 0.28 |
| rs116125333 | 2.44E-06 | 1 |
| rs11648436 | 1.77E-07 | 1 |
| rs117132860 | 7.17E-06 | 0.112 |
| rs117204628 | 2.24E-06 | 0.84 |
| rs11739906 | 1.14E-10 | 1 |
| rs117462393 | 9.02E-07 | 1 |
| rs12203592 | 9.80E-06 | <0.056 |
| rs12350739 | 4.87E-09 | 1 |
| rs1267037 | 1.15E-08 | 1 |
| rs1278766 | 2.41E-07 | 1 |
| rs12913832 | 4.65E-08 | 1 |
| rs1308048 | 3.26E-07 | 0.784 |
| rs1338549 | 9.03E-10 | 1 |
| rs1391659 | 5.08E-08 | 1 |
| rs1437635 | 7.08E-07 | 0.28 |
| rs150527451 | 5.02E-07 | 1 |
| rs1548714 | 6.82E-07 | 0.168 |
| rs1584885 | 1.27E-07 | 1 |
| rs186425026 | 4.30E-08 | 1 |
| rs2493411 | 4.02E-09 | 1 |
| rs251466 | 1.09E-09 | 1 |
| rs2518796 | 4.70E-08 | 1 |
| rs2737217 | 6.03E-07 | 0.056 |
| rs3114908 | 1.42E-06 | <0.056 |
| rs3213737 | 2.37E-07 | 1 |
| rs35407 | 3.38E-06 | 1 |
| rs4098 | 3.88E-07 | 0.392 |
| rs4406278 | 8.90E-07 | <0.056 |
| rs4578351 | 8.23E-08 | 1 |
| rs4670813 | 5.29E-08 | 1 |
| rs59036912 | 8.02E-07 | 0.728 |
| rs6007506 | 1.13E-08 | 1 |
| rs61813875 | 8.47E-08 | 1 |
| rs62209647 | 4.70E-06 | <0.056 |
| rs6689641 | 7.92E-08 | 1 |
| rs67279079 | 1.38E-07 | 1 |
| rs6882046 | 1.16E-07 | 1 |
| rs6949073 | 6.82E-07 | 0.056 |
| rs7034354 | 3.81E-09 | 1 |
| rs7197795 | 8.65E-07 | 1 |
| rs731135 | 3.75E-07 | 0.616 |
| rs7768317 | 1.34E-07 | 1 |
| rs849138 | 9.46E-08 | 1 |
| rs9832130 | 2.93E-07 | 0.952 |
| rs9835772 | 9.82E-08 | 1 |
| rs9858244 | 3.04E-08 | 1 |
| rs9926609 | 1.58E-07 | 1 |

supplementary table3 Inverse variance weighted (fixed effects) results

| outcome | exposure | method | nsnp | b | se | pval |
| --- | --- | --- | --- | --- | --- | --- |
| Sunburn | CM | Inverse variance weighted (fixed effects) | 49 | 0.025843 | 0.001721 | 5.54E-51 |
